# A new NASH model in aged mice with rapid progression of steatohepatitis and fibrosis

**DOI:** 10.1371/journal.pone.0286257

**Published:** 2023-05-25

**Authors:** Xuecheng Li, Yi Lu, Xiaoshuang Liang, Xiaofei Zhou, Dirui Li, Zan Zhang, Yunchao Niu, Shuaishuai Liu, Ling Ye, Rufeng Zhang

**Affiliations:** Biocytogen Pharmaceuticals (Beijing) Co., Ltd, Beijing, Daxing District, China; Texas A&M University, UNITED STATES

## Abstract

Non-alcoholic fatty liver disease (NAFLD) has a high prevalence worldwide, with a significant proportion of patients progressing into non-alcoholic steatohepatitis (NASH) and further into cirrhosis and hepatocellular carcinoma (HCC). Most of the current animal models of NASH have limitations, such as incompatibility with human pathogenesis characteristics or long induction periods, which severely limit the development of new drugs and preclinical studies for NASH. We investigated the progression of NASH and fibrosis, as well as metabolic indicators, at different time points in aged mice induced by the Gubra Amylin NASH (GAN) diet, a high-fat, high-sugar, high-cholesterol diet, and attempted to establish a rapid and useful mouse model of NASH. Young and aged C57BL/6 mice were induced on a normal chow or GAN diet for 12 and 21 weeks, respectively. After 12 weeks of induction, aged mice developed NASH, including hepatic steatosis, lobular inflammation and hepatic ballooning, and the phenotype was more severe compared with young mice. After 21 weeks of induction, aged mice developed hepatic fibrosis, which greatly shortened the induction time compared with young mice. Furthermore, analysis of immune cell infiltration in the liver by flow cytometry elucidated the changes of multiple immune cells during the pathogenesis of NASH. These findings suggest that aged mice may develop NASH and fibrosis more rapidly under GAN diet induction, which may significantly shorten the period for preclinical studies of NASH.

## Introduction

Non-alcoholic fatty liver disease (NAFLD) is one of the leading causes of chronic liver disease worldwide. In the United States and Europe, NAFLD will overtake hepatitis C as the leading cause of liver transplantation [1,2]. There are currently no drugs approved by the FDA, EMA or NMPA for the treatment of NASH. Approximately 30% of adults have non-alcoholic fatty liver disease (NAFLD) [3], of which approximately 15% progress to non-alcoholic steatohepatitis (NASH) [4], and then to cirrhosis and hepatocellular carcinoma (HCC) [5]. The progressive phase of NASH is usually accompanied by fibrosis in addition to features such as hepatocellular steatosis, lobular inflammation and hepatocyte ballooning [6]. The causes of NASH are complex and one of the most important aspects is the metabolic syndrome characterized by obesity and insulin resistance [7]. Under the accumulation of liver fat and insulin resistance, if inflammation is involved, it causes a second hit in the pathogenesis of NASH, which is the classic ’two hit’ hypothesis of NASH pathogenesis [8]. It is now theorized that multiple factors such as metabolic disorders, gut microbiota leakage and genetic and epigenetic factors cause liver inflammation, fibrosis formation, necrosis and hepatocyte apoptosis, which eventually develop into cirrhosis and HCC [9–12].

Appropriate animal models are essential for the study of disease pathogenesis and drug development. Due to the complexity of NASH disease, it is difficult to find a perfect approach that mimics all the characteristics of NASH patients. Current animal models of NASH can be divided into three main categories: genetically modified animal models, diet-induced animal models and chemically induced animal models, on the basis of which certain combinations can be applied [13–15]. The most commonly used genetic models are the classic obese and diabetic mouse models, including db/db mice and ob/ob mice. Among the diet-induced models of NASH, the high-fat, high-sugar, high-cholesterol (HFHC) diet induced NASH model is well known, which is closer to the human disease profile and usually requires a longer induction period [16]. AMLN diet or a modified AMLN diet (GAN diet, replacing Primex Shortening with equal amounts of palm oil) is a class of diets with high levels of fat, fructose, and cholesterol that has been well studied in the NASH model of induction [17]. It has also been widely used in preclinical drug efficacy evaluation [18,19]. GAN diet induces obesity, insulin resistance, hepatic steatosis and inflammation in mice with disease characteristics that are highly similar to those of human NASH patients [20].

The other is the nutrient-deficient dietary models, such as choline-deficient (CD), methionine-choline deficient (MCD), and choline-deficient, l-amino acid-defined (CDAA). This category of diets can induce more severe NASH features within 6–8 weeks and is accompanied by fibrosis, but the mice in this model lose weight and the mechanism of pathogenesis is less consistent with the clinical features [21,22]. In chemical induction models, carbon tetrachloride is one of the most commonly used induction reagents. The metabolites of carbon tetrachloride in the liver cause oxidative damage and activate hepatic stellate cells, leading to inflammation and fibrosis in the liver [23].

In this study, the aged mice supplemented with high-fat, high-sugar, high-cholesterol diet were used for the first time in the construction of NASH model, in order to simulate the development process of human diseases rapidly, providing convenience for the preclinical research and drug development of NASH.

## Materials and methods

### Animals

Healthy young male mice, aged male mice, and aged female mice on C57BL/6 background were provided by Biocytogen Pharmaceuticals (Beijing) Co., Ltd. All animal studies were performed according to the protocol approved by the Animal Care and Use Committee of Biocytogen Pharmaceuticals. The experimental animals were housed in the SPF barrier facility and given food and water *ad libitum* with a controlled temperature (22 ± 2°C), a 12 h/12 h light/dark cycle. At the end of the experiment, mice were euthanized by CO_2_ inhalation.

For the diet-induced NASH model, mice at 5 weeks of age (young mice) or 56 weeks of age (aged mice) were induced by feeding a Gubra-Amylin NASH Diet (Research Diets, D09100310, 40% fat kcal, 22% fructose and 2% cholesterol) or a normal chow diet for 12,16 and 21 weeks.

### Serum analysis

The whole blood was obtained from the inner canthus in mice, and centrifuged at 3000 rpm for 10 min after standing at room temperature for 20 minutes to separate the serum.

Aspartate transaminase (AST) and alanine transaminase (ALT), triglycerides (TG), and total cholesterol (TC) in the serum were measured using an automatic biochemistry apparatus (Hitachi *3100*, HITACHI, Tokyo, Japan) and corresponding assay kits.

### Histology analysis

The mice were euthanized and the livers were removed, fixed in 10% formalin and stored in fixative overnight. Formalin-fixed liver sections were embedded in paraffin and sectioned at 4μm. HE staining was performed using an HE staining kit (Beyotime, Beijing, China) according to the manufacturer’s instructions. Histological assessment and scoring were performed by a pathologist blinded to the study. The NAFLD activity score (NAS) is the sum of the steatosis, lobular inflammation and hepatocellular ballooning scores. Sirius red staining was performed using the Picro-Sirius red staining kit (G-CLONE, Beijing, China) according to the manufacturer’s instructions.

Immunohistochemical analysis was performed on paraffin-embedded liver sections stained with α-SMA antibody (Abcam, Cambridge, MA, USA). Quantitative analysis of immunohistochemical staining was performed using HALO image analysis software (HALO™, Indica Labs, Inc., Corrales, NM, USA).

### IPGTT

The intraperitoneal glucose tolerance test (IPGTT) was performed 12 weeks after dietary induction; mice were fasted overnight before the day of the glucose tolerance test. After measuring baseline blood glucose levels, mice were injected intraperitoneally with glucose (2 g/kg body weight) and blood glucose was measured at 15, 30, 60, 90 and 120 min after the glucose challenge using an AccuCheck blood glucose meter (Roche, Basel, Switzerland). The glucose area under the curve (AUC) during a GTT from 0 to 120 min was calculated and analyzed using GraphPad Prism 7.

### Flow cytometry

Liver tissues were cut up, digested with collagenase type IV (Roche, Basel, Switzerland); submitted in GentleMACS Octo dissociator (Miltenyi Biotec) tissue processor, and then pressed through 70 mm cell strainers to achieve single cell suspensions. Red blood cell lysis was performed using Red Blood Cell Lysis Solution (Beyotime, Shanghai, China). Multi-colour staining was conducted using combinations of the following monoclonal antibodies: CD45, CD115, CD3, CD4, CD8, F4/80, CD11b, Ly6C, Ly6G, (all BioLegend, San Diego, CA, USA). Dead cells were excluded by Fixable Viability Dye eFluor™ 506 (eBioscience, San Diego, CA USA). Flow-cytometric analysis was performed on aAttune NxT Flow Cytometer (Thermo Fisher, Waltham, MA, USA) and analysed with FlowJo (Tree Star, Ashland, OR USA).

### Statistical analysis

Data are expressed as means ± SEM. Statistical significance was analyzed by two-tailed Student’s t-test. A value of P<0.05 was considered statistically significant.

## Results

### Metabolic dysfunction of aged mice

In this study, we used the GAN diet to induce young male mice, aged male mice and aged female mice for different durations and then investigated body weight, blood glucose, lipids, blood biochemistry, glucose tolerance and insulin levels of mice at different time periods under chow and GAN diet induction conditions.

**Fig 1:**
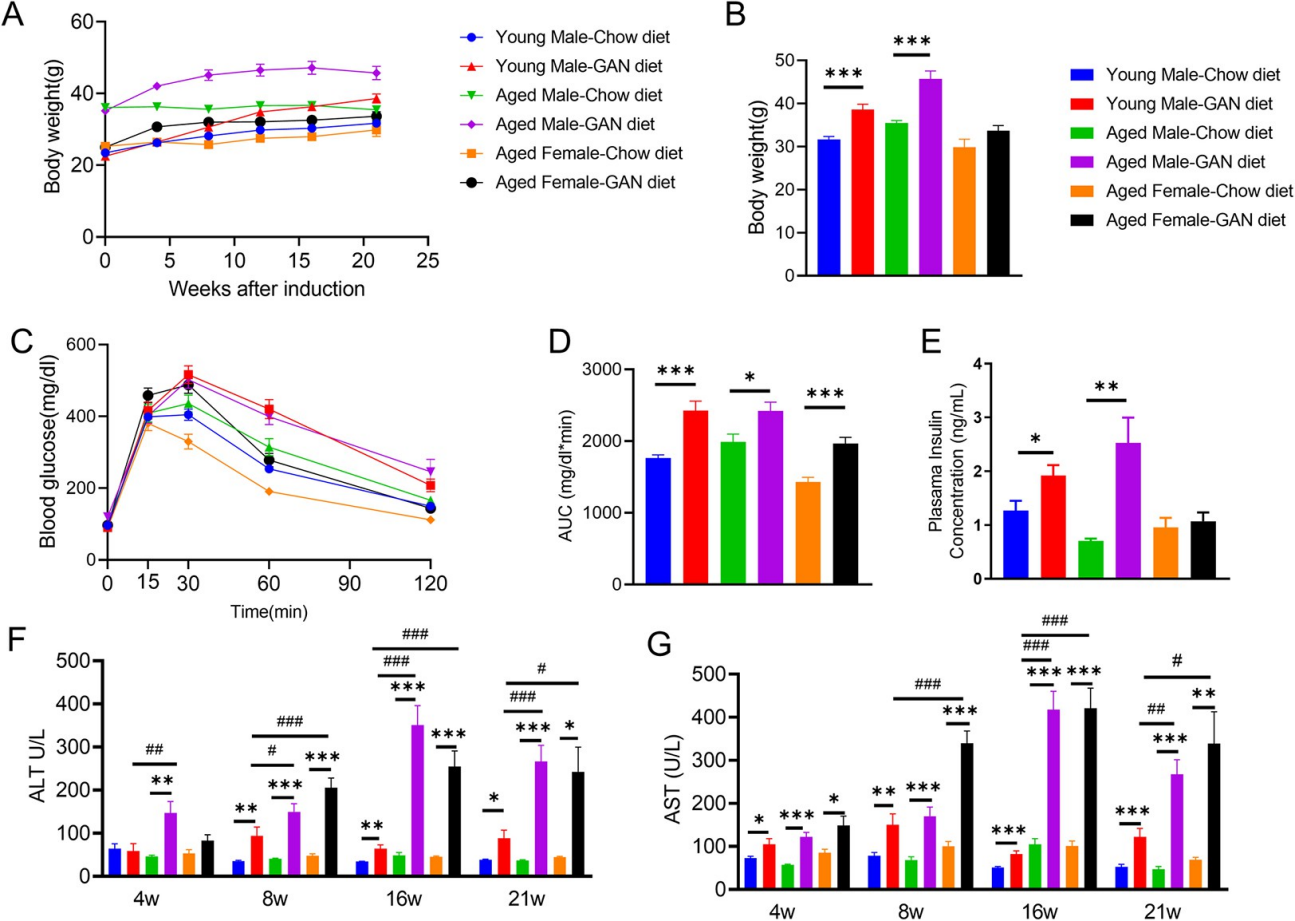
Metabolic profile of mice after GAN diet induction. (A-B) Increased body weight after GAN diet induction. (C) Impaired glucose tolerance ability induced by Gubra-Amylin diet. (D) Area under curve of **C**. (E) Increased plasma insulin content in Gubra-Amylin diet induction group. (F-G) Increased ALT(F) and AST(G) in Gubra-Amylin diet induction group. Data are means ±SEM. N = 6–30 mice per group. *P <0.05, **P <0.01, ***P <0.001, #P <0.05, ##P <0.01, ###P <0.001.

First, under GAN diet induction, young male mice gradually gained weight, reaching 38.6 g at 21 weeks of induction, and aged males reached a weight of about 45 g after 8 weeks, after which they remained relatively stable until 21 weeks. Aged female mice showed relatively low weight gain, reaching around 33.7g at 21 weeks of induction (Fig 1A and 1B). Blood glucose levels of both young and aged male mice increased over time under GAN diet-induced conditions, while aged females showed a slight increase, but to a lesser extent than males (S1A–S1C Fig).

To further understand the metabolic profile of the mice, we performed glucose tolerance tests after 12 weeks of induction and found that young and aged male mice showed similar levels of impaired glucose tolerance, while aged females showed reduced levels of impaired glucose tolerance compared with the corresponding male groups (Fig 1C and 1D). We also measured plasma insulin levels and found that both young and aged male mice showed a significant increase in insulin levels after induction of the GAN diet compared with chow conditions. In contrast, there was no significant change in aged female mice (Fig 1E).

Blood biochemistry results showed that serum ALT and AST levels in these models also increased progressively up to 16 weeks of induction and decreased at 21 weeks. ALT and AST levels of aged mice were higher than those of young mice at all time points (Fig 1F and 1G).

Serum cholesterol also showed significant increases at different time points after induction in both young and aged mice, but the increase was relatively small in the aged female induction group (S1D and S1E Fig).

### Aged mice accelerate GAN diet-induced steatohepatitis

**Fig 2:**
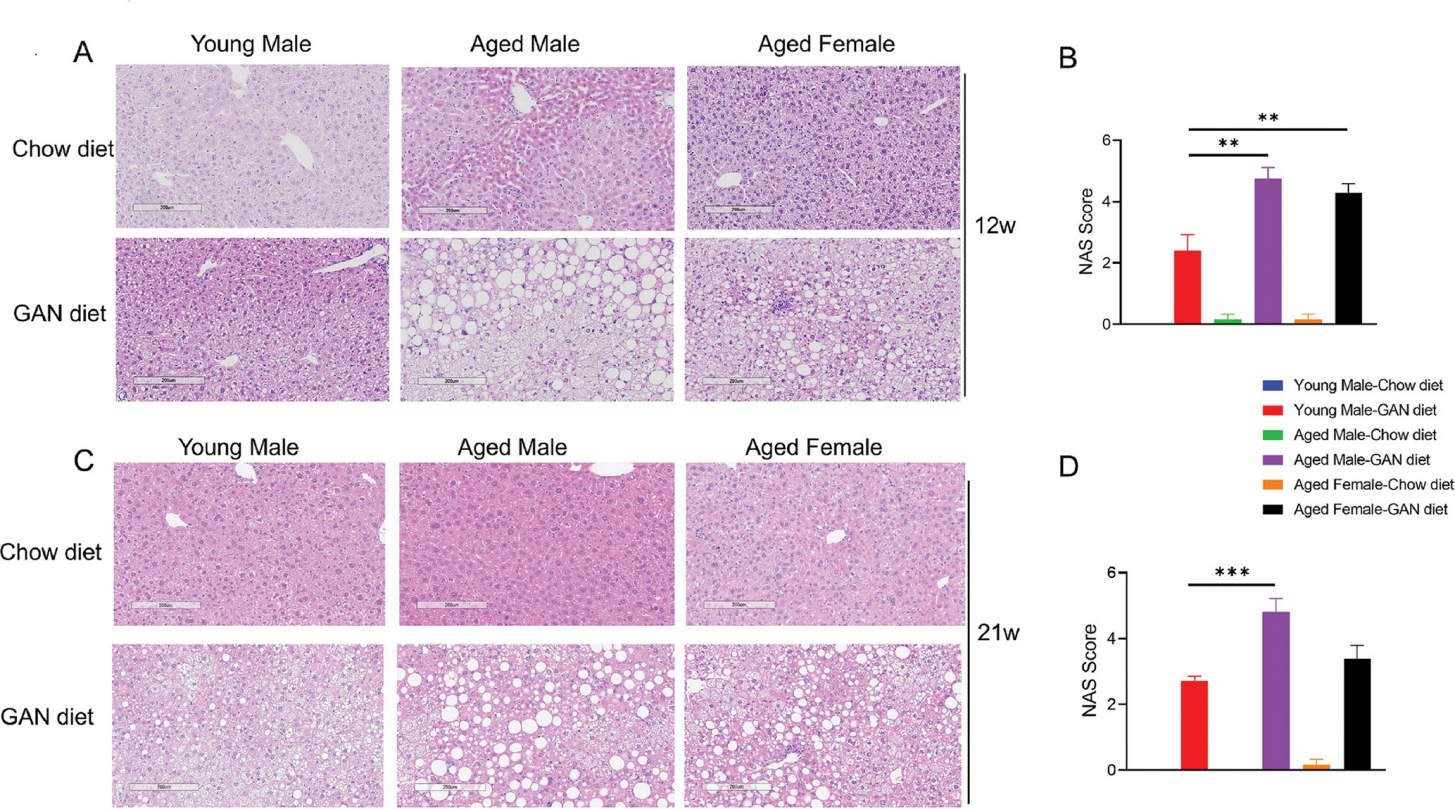
GAN diet-induced Nonalcoholic Steatohepatitis in young and aged mice. (A) Representative pictures of H&E staining after GAN diet induction for 12 weeks. (B) NAFLD activity score (NAS) of different groups after 12 weeks induction. (C) Representative pictures of H&E staining after GAN diet induction for 21 weeks. (D) NAFLD activity score (NAS) of different groups after 21 weeks induction. N = 6–10 mice per group. Data are expressed as mean ± SEM. *P <0.05, **P <0.01, ***P <0.001.

We used the NAS scoring system for NAFLD to calculate the activity scores of NAFLD in each group of mice. It was found that aged male mice induced with the GAN diet for 12 weeks exhibited a more pronounced NASH phenotype, including hepatic steatosis, inflammatory infiltration and ballooning, and their NAS scores were significantly higher compared with the younger male induction group. At the same time, we found that aged female mice also exhibited more severe NASH symptoms after 12 weeks of induction (Fig 2A and 2B). After 21 weeks of induction on the GAN diet, aged male mice still exhibited a more severe NASH phenotype (Fig 2C and 2D). In contrast, few aged male and female mice developed spontaneous steatosis and intralobular inflammation under chow conditions (Table 1).

**Table 1:** NAFLD activity score (NAS) of different groups after 12 or 21 weeks of induction.

**12 weeks**				
**Parameter**	**Steatosis**	**Lobular Inflammation**	**Hepatocyte Ballooning**	**NAS**
Young Male-Chow diet	0	0	0	0
Young Male-GAN diet	1.3±0.2***	0.6±0.2*	0.6±0.2*	2.5±0.3***
Aged Male-Chow diet	0.2±0.2	0	0	0.2±0.2
Aged Male-GAN diet	2.9±0.1*** ^###^	1.0±0.3*	0.8±0.2*	4.7±0.4*** ^##^
Aged Female-Chow diet	0	0.2±0.2	0	0.2±0.2
Aged Female-GAN diet	2.7±0.2*** ^###^	0.6±0.2	0.9±0.1***	4.2±0.2*** ^##^
**21 weeks**				
**Parameter**	**Steatosis**	**Lobular Inflammation**	**Hepatocyte Ballooning**	**NAS**
Young Male-Chow diet	0	0	0	0
Young Male-GAN diet	1.7±0.2***	0.6±0.2*	0.6±0.2*	2.9±0.3***
Aged Male-Chow diet	0	0	0	0
Aged Male-GAN diet	2.7±0.2*** ^###^	1.1±0.3*	1.0±0.1***	4.8±0.4*** ^###^
Aged Female-Chow diet	0	0.2±0.2	0	0.2±0.2
Aged Female-GAN diet	2.1±0.2	0.8±0.3	0.8±0.2**	3.7±0.4***

NAFLD activity score (NAS) was calculated according to the method described by Kleiner et al [24]. Data are mean±SEM (N = 6–10 mice per group). Significance was determined using the Student’s *t*-test.

* p<0.05

** p<0.01

*** p<0.001 *versus* sex and age matched Chow diet.

^#^ p<0.05

^##^ p<0.01

^###^ p<0.001 *versus* Young Male-GAN diet group.

### Aged mice accelerate immune cell infiltration of the liver

**Fig 3:**
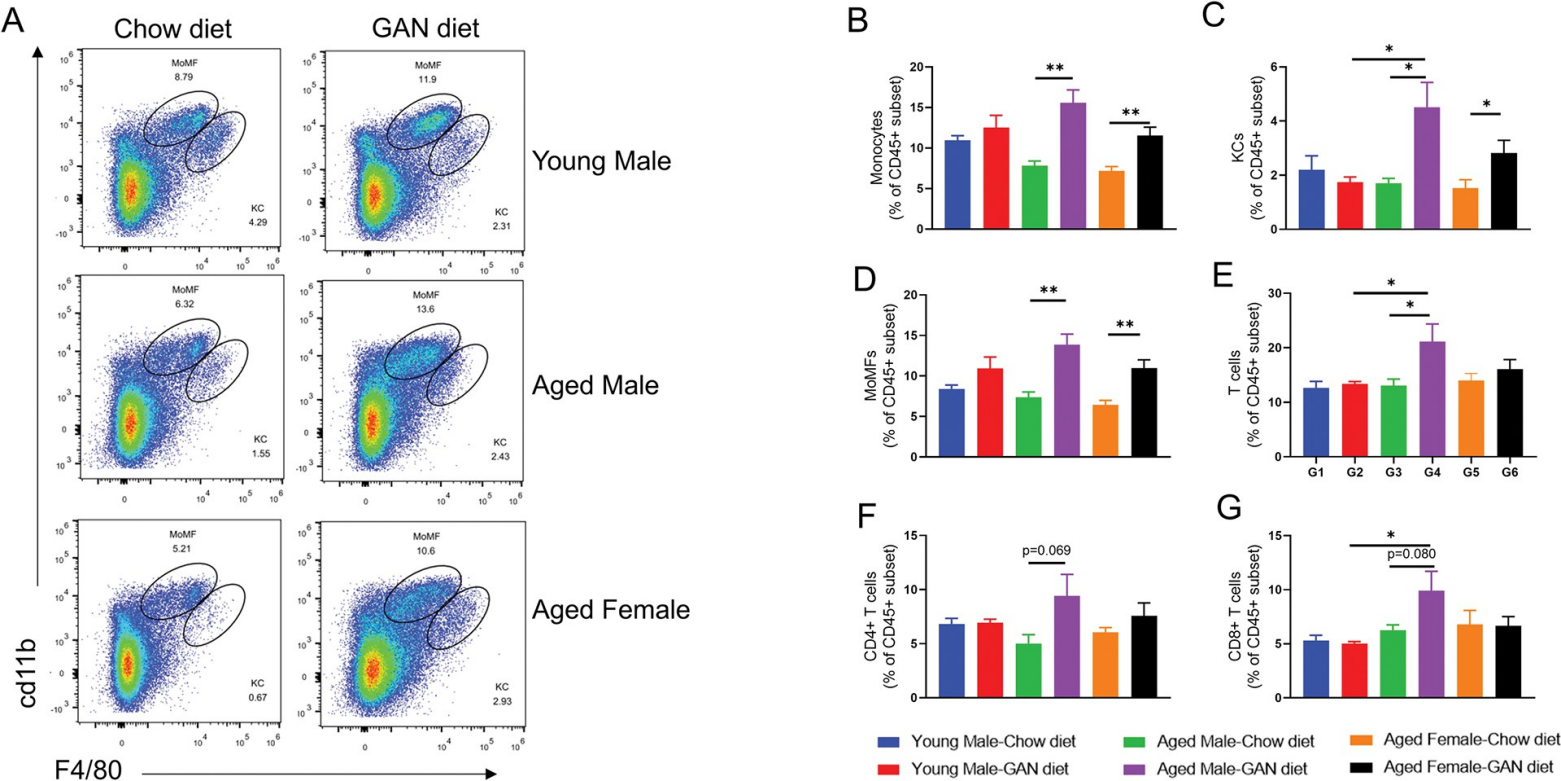
Immune cell infiltration of the liver after GAN diet induction for 12 weeks. (A) Percentage of infiltrating monocytes (CD11b^int^F4/80^low^) and Kupffer cells (CD11b^+^F4/80^hi^) in liver assessed by the flow cytometry. (B-G) Analysis of monocytes(B), KCs (C), MoMFs (D), T cells (E), CD4+ T cells (F) and CD8+ T cells (G) in the liver. N = 6–10 mice per group. Data are expressed as mean ± SEM. *P <0.05, **P <0.01, ***P <0.001.

**Fig 4:**
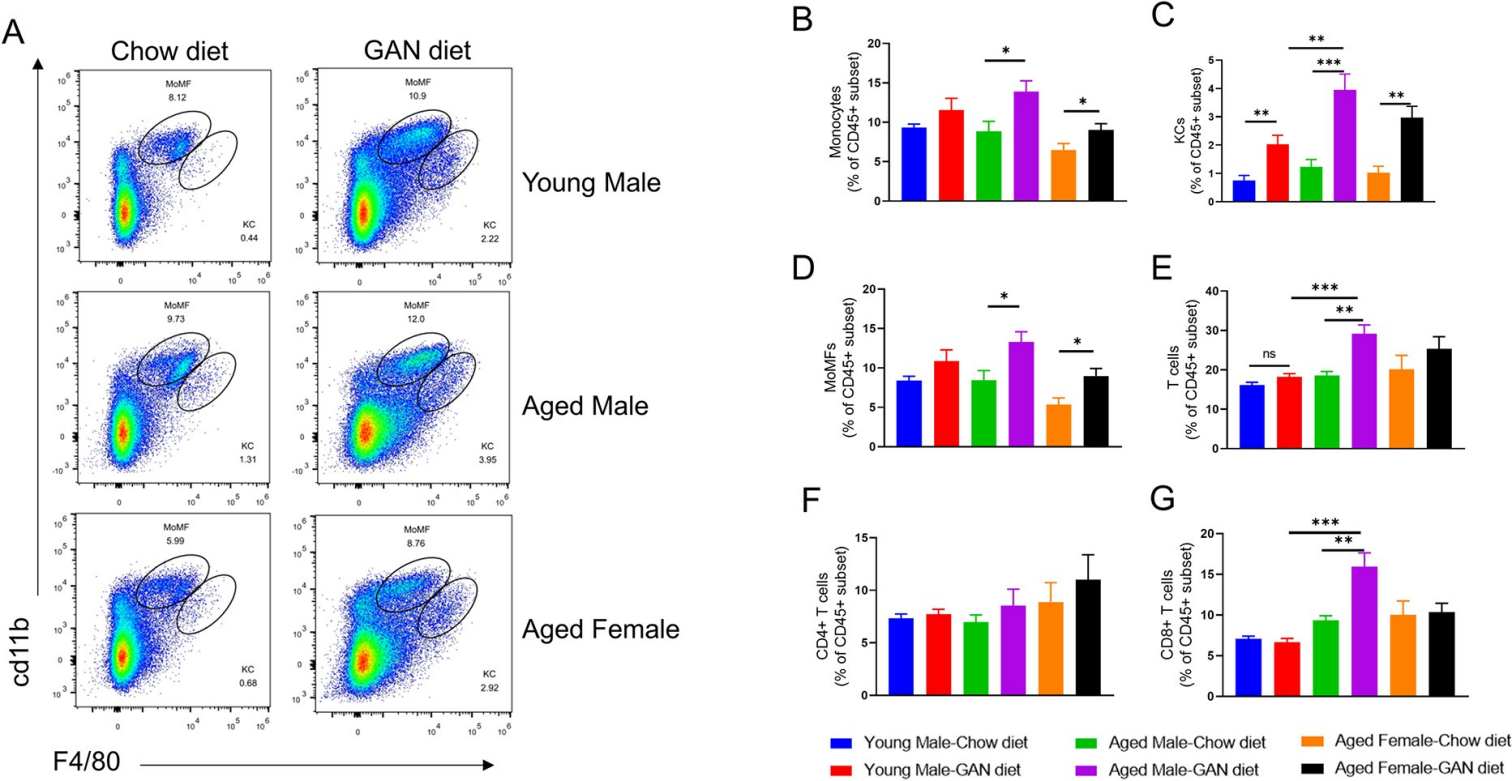
Immune cell infiltration of the liver after GAN diet induction for 21 weeks. (A) Percentage of infiltrating monocytes (CD11b^int^F4/80^low^) and Kupffer cells (CD11b^+^F4/80^hi^) in liver assessed by the flow cytometry. (B-G) Analysis of monocytes(B), KCs (C), MoMFs (D), T cells (E), CD4+ T cells (F) and CD8+ T cells (G) in the liver. N = 6–10 mice per group. Data are expressed as mean ± SEM. *P <0.05, **P <0.01, ***P <0.001.

Immune cell infiltration is an important feature of NASH, where innate immune cells, represented by macrophages, and adaptive immune cells, represented by T cells, are considered to be the two major cell types involved in the formation of inflammatory foci. We analyzed immune cell infiltration in the liver by flow cytometry and found that there was a significant increase in monocytes and monocyte-derived macrophages (MoMFs) in both the aged male and aged female induction groups, but not in the young male induction group. Meanwhile, Kupffer cells (KCs) were significantly increased in the induction group at all ages (Fig 3A–3D). These immune cells showed similar results after 21 weeks of induction as after 12 weeks (Fig 4A–4D).

The proportion of total T cells in the GAN diet group of aged male mice after 12 weeks of induction was significantly higher than in the Chow diet group of aged male mice and the GAN diet group of young male mice (Fig 3E), the proportion of CD8 T cells and CD4 T cells tended to increase in the GAN diet group of aged male mice compared with the chow diet group of aged male mice, while there was a significant increase in CD8 T cells compared with the GAN diet group of young male mice (Fig 3F and 3G). No significant changes in the corresponding cell proportions were observed in aged female mice or young male mice under GAN diet induction compared with chow diet. Analysis of the groups of mice at 21 weeks of induction also showed that the most significant changes in total T cells as well as CD8+ T cells were found in the aged male mice induction group (Fig 4E–4G).

### Aged mice accelerate liver fibrosis and promote activation of HSCs

**Fig 5:**
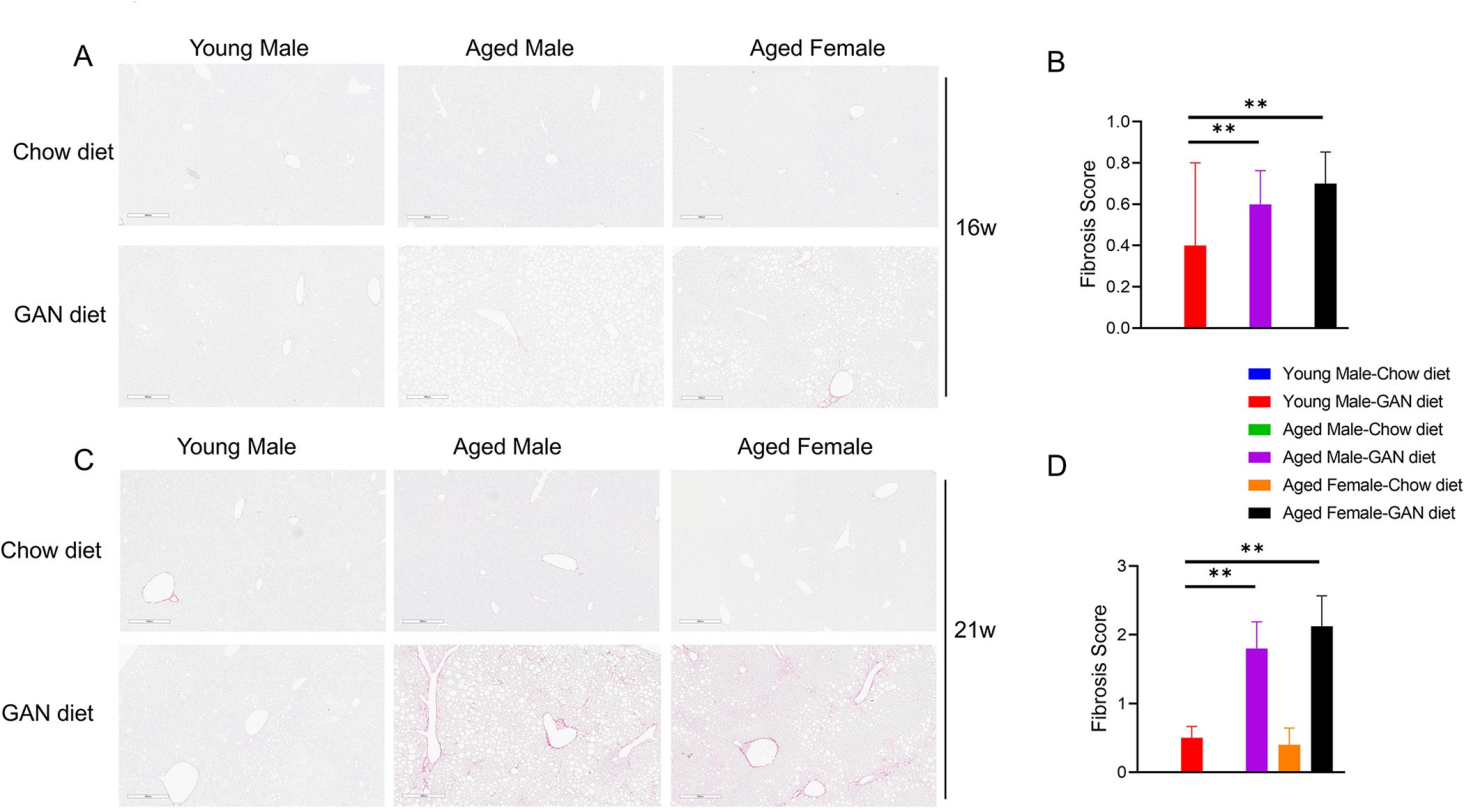
GAN diet-induced fibrosis in young and aged mice. (A) Representative pictures of Sirius Red staining after GAN diet induction for 16 weeks. (B) Fibrosis score of mice after GAN diet induction for 16 weeks. (C) Representative pictures of Sirius Red staining after GAN diet induction for 21 weeks. (D) Fibrosis score of mice after GAN diet induction for 21 weeks. N = 6–10 mice per group. Data are expressed as mean ± SEM. *P <0.05, **P <0.01, ***P <0.001.

**Fig 6:**
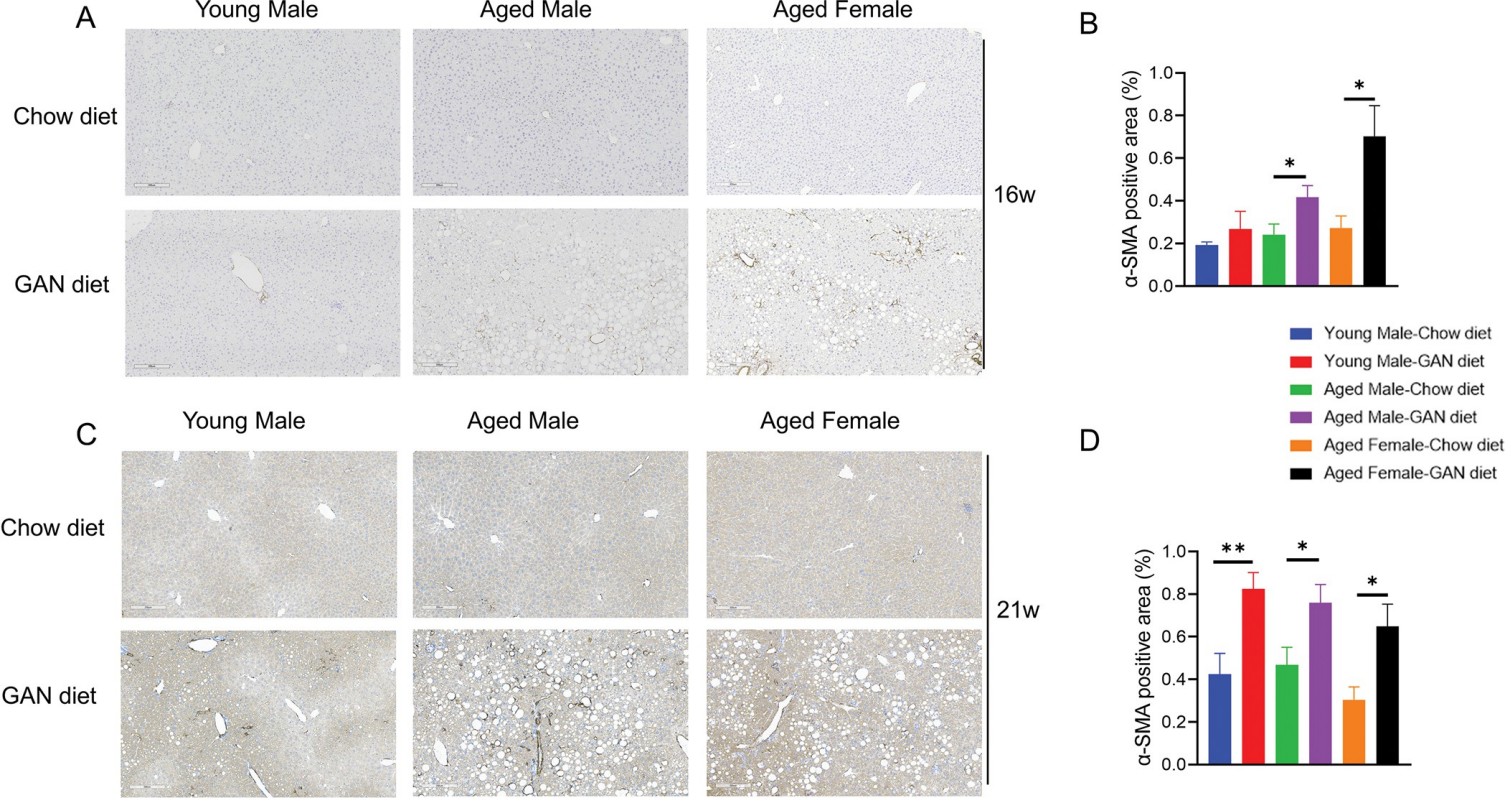
**Activation of hepatic stellate cells** (A) Immunostaining for αSMA in liver sections from representative mice after GAN diet induction for 16 weeks. (B) Quantification of αSMA-positive tissue area after GAN diet induction for 16 weeks. (C) Immunostaining for αSMA in liver sections from representative mice after GAN diet induction for 21 weeks. (D) Quantification of αSMA-positive tissue area after GAN diet induction for 21 weeks. N = 6–10 mice per group. Data are expressed as mean ± SEM. *P <0.05, **P <0.01, ***P <0.001.

Liver fibrosis is an important turning point in the progression of a healthy liver to cirrhosis. Stimulated by various inflammatory factors, hepatic stellate cells (HSCs) secrete extracellular matrix upon activation, leading to liver fibrosis. Sirius red staining showed that after 16 weeks of induction, young and aged mice all showed a very mild degree of fibrosis (Fig 5A and 5B). After 21 weeks of induction, young mice still showed a mild degree of fibrosis. In contrast, both male and female aged mice showed a significant increase in liver fibrosis (Fig 5C and 5D). Immunohistochemical staining for α-SMA, a marker of HSC activation, showed an earlier increase in positive signals in the induction groups of aged male and aged female mice compared with young mice after 16 weeks of induction (Fig 6A and 6B). And after 21 weeks of induction, the α-SMA signal reached higher levels in all induction groups (Fig 6C and 6D).

## Discussion

In the present study, we induced a high-fat, high-sugar, high-cholesterol diet in aged mice and found that 12 weeks after induction, aged male mice showed significant NASH features, including steatosis, intralobular inflammation and hepatocyte ballooning, which were more pronounced than in young male mice under the same conditions. The main advantage of this model is that it induces human-like pathological features in mice in a short time. On the other hand, most preclinical studies of metabolic diseases use male mice, and more attention should be paid to female mice in drug trials. Although a variety of NASH induction methods have been widely used, there are still limitations. In young C57BL/6 mice, induction with a high-fat, high-sugar, high-cholesterol diet can mimic most of the features of human NASH disease, but it usually requires a long period of time and the degree of fibrosis is not obvious [19,25,26]. Although nutritional-deficient dietary models can rapidly mimic NASH and fibrotic features, the metabolic profile of such NASH models is distinct from that of human NASH [27].

An important hallmark of NAFLD progression to the NASH stage is the infiltration of immune cells in the liver. These immune cells consist of two main types, innate immunity and adaptive immunity, respectively [28]. In the Innate immune responses, Kupffer cells are able to respond directly to external stimuli such as excess free fatty acids, cholesterol and LPS [29,30] and then release cytokines and chemokines such as TNFα and CCL_2_ [31,32].

Chemokines released from Kupffer cells can further recruit other immune cells to the liver, such as monocytes, neutrophils, natural killer (NK) and natural killer T (NKT) cells [30,33], with monocyte-derived macrophages (MoMFs) being the most important and best studied immune cell type upon stimulation. Recruitment of MoMFs to the liver is dependent on CCR2, CX3CR1 and CXCR3 [15,34,35], among others, followed by differentiation into a class of macrophages with high Ly6C expression, which can further promote inflammation and activate hepatic stellate cells leading to fibrosis progression. Targeting macrophages is therefore also an important strategy for the treatment of NASH [36].

Lymphocytes infiltration is another important feature of the NASH process, which has been well studied in NASH patients as well as in mouse models of NASH [37,38]. CD4+ T cells can further differentiate into T helper 1(TH1), T helper 2(TH2), T helper 17(TH17) and Treg, of which TH1 and TH17 are the most studied, have been shown to promote the development of NASH, and blocking the corresponding signaling pathways can alleviate the progression of NASH in murine models [39,40]. CD8+ cytotoxic T cells are also an important component of immune cells in NASH. CD8+ T cells are recruited in response to IFNα-mediated signaling and are able to promote insulin resistance and hepatic glucose metabolism in a high-fat diet mouse model. In a choline-deficient high-fat diet-induced NASH model, *β2m*^-/-^ mice deficient in CD8+ T cells and NK cells are protected against steatosis and inflammatory cell infiltration. Selective ablation of CD8+ T cells also protects against the high-fat, high- carbohydrate (HF-HC) diet-induced NASH phenotype in mice [38,41]. Our results showed that GAN diet-induced aged male mice exhibited an earlier and more pronounced immune cell infiltration in the liver than young males. Specifically, a significant increase in Kupffer cells, MoMFs and T cells was detected in the liver of aged male mice by 12 weeks of induction, whereas only an increase in the proportion of Kupffer cells was detected in young male mice up to 21 weeks of induction.

Another important feature of our NASH model is the development of liver fibrosis. Liver fibrosis is an intermediate stage in the progression from NASH to cirrhosis. Hepatic stellate cells are known to be the main driver of liver fibrosis, in response to various cytokines, chemokines, etc., hepatic stellate cells are activated and then secrete extracellular matrix, leading to the accumulation of liver fibrosis. Our results show that in aged mice, significant liver fibrosis can develop after 21 weeks of induction, a phenomenon that has been difficult to achieve in previous animal models without the addition of CCL_4_ induction. Given that the FDA sets the endpoints for clinical trials include: NASH improvement without worsening of fibrosis and fibrosis improvement without worsening of NASH, our study provides a highly translatable, time-saving NASH model for preclinical drug development.

There are also limitations in our NASH model. First, although the current study illustrates that geriatric mouse induction can be a better model for NASH, the model needs some NASH clinical drugs for further validation, such as GLP1R agonists [18], THR-β agonists [19] and FGF21 analogs [42]. Secondly, we did not observe hepatocellular carcinoma development in mice after 21 weeks of induction; whether hepatocellular carcinoma will occur with a longer induction time remains to be investigated. As the proportion of hepatocellular carcinoma caused by NASH is increasing, if this NASH model can induce hepatocellular carcinoma in mice more rapidly, it will play a more important role in the development of drugs for liver disease.

## Supporting information

S1 Fig Metabolic profile of mice after GAN diet induction. (A-C) Increased blood glucose of young male mice (A), aged male mice (B) and aged female mice (C) after GAN diet induction. (D) Triglyceride change after GAN diet induction. (E) Total cholesterol change after GAN diet induction. N = 10–30 mice per group. *P <0.05, **P <0.01, ***P <0.001.
(PDF)
