# Supplementary figures and images for: A new NASH model in aged mice with rapid progression of steatohepatitis and fibrosis
Source: PLoS One. 2023 May 25;18(5):e0286257. doi: 10.1371/journal.pone.0286257 (PMC10212180; doi:10.1371/journal.pone.0286257)

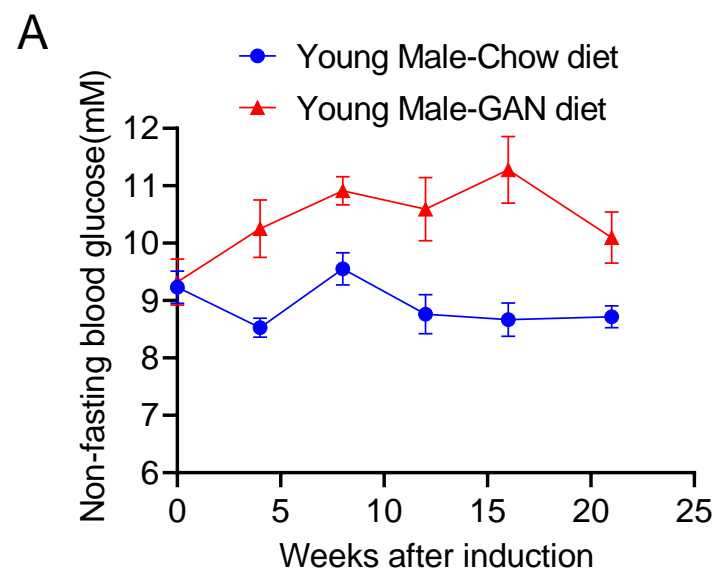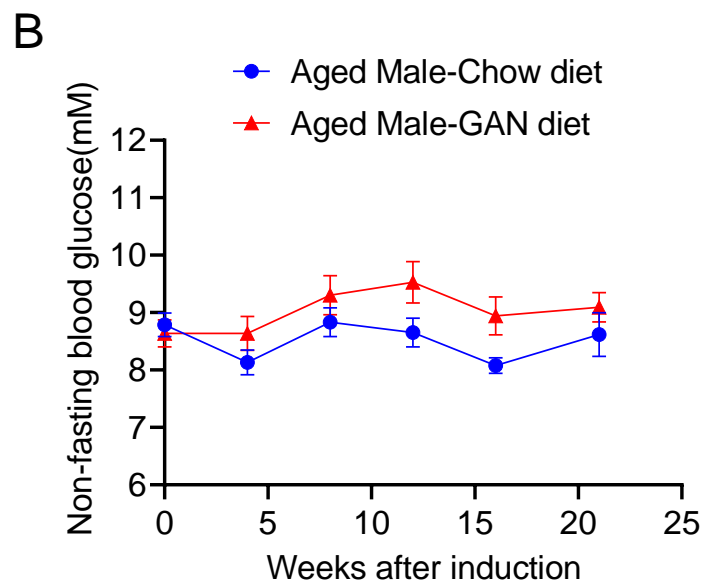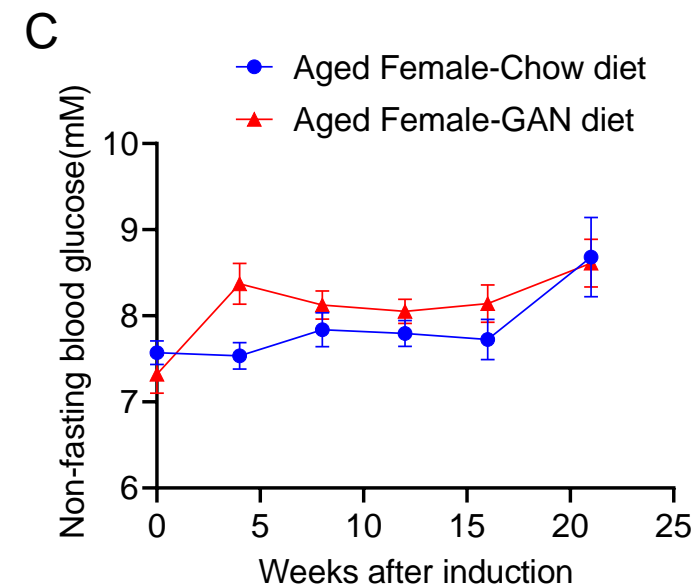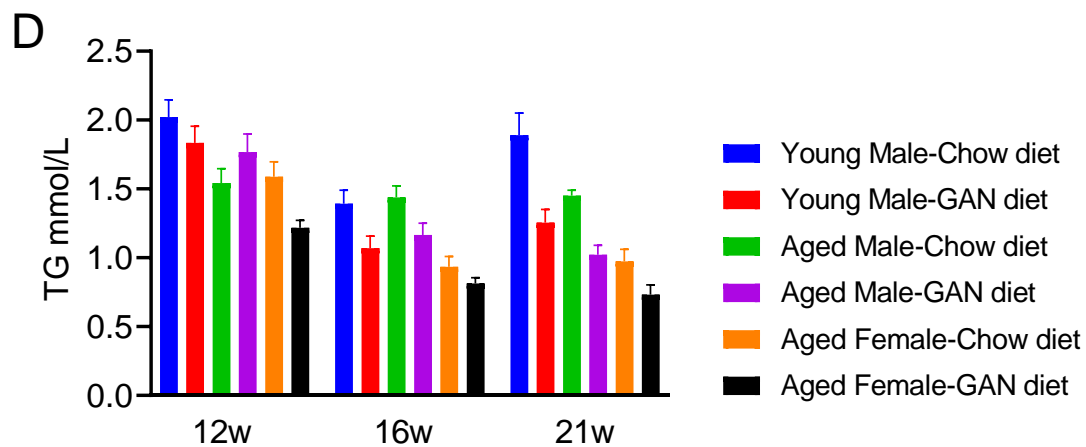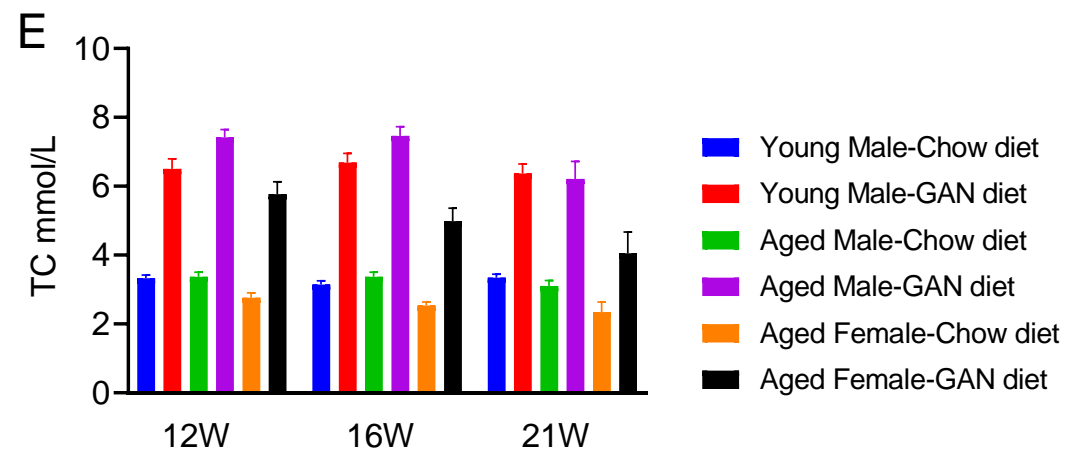

Supplement: S1 Fig — (A-C) Increased blood glucose of young male mice (A), aged male mice (B) and aged female mice (C) after GAN diet induction. (D) Triglyceride change after GAN diet induction. (E) Total cholesterol change after GAN diet induction. N = 10–30 mice per group. *P <0.05, **P <0.01, ***P <0.001. (PDF) [file pone.0286257.s001.pdf]
